# Characterization of the complete chloroplast genome of *Arachis pintoi* Krapov. & W.C.Greg., a perennial leguminous forage

**DOI:** 10.1080/23802359.2021.1981786

**Published:** 2021-11-23

**Authors:** Xiao-Li Zhang, Ling-Long Zhu, Du-Lin Song, Fu-Zhen Li

**Affiliations:** Institute of Crop and Nuclear Technology Utilization, Zhejiang Academy of Agricultural Sciences, Hangzhou, P. R. China

**Keywords:** *Arachis pintoi*, Leguminosae, chloroplast genome, phylogenetic analysis

## Abstract

*Arachis pintoi* Krapov. & W.C.Greg. is an important leguminous forage grass species that have extremely wide ranges of distribution in the tropical and sub-tropical regions. It has high feeding value and horticultural value. In this study, we sequenced and assembled the complete chloroplast genome of *A. pintoi*. The chloroplast genome is 156,185 bp in length, containing a pair of inverted repeated (IR) regions of 25,820 bp that are separated by a large single copy (LSC) region of 85,637 bp, and a small single copy (SSC) region of 18,908 bp. The complete chloroplast genome contains 112 unique genes, including 80 protein-coding genes, 28 transfer RNA genes (tRNAs), and four ribosomal RNA genes (rRNAs). The overall GC content was 36.4%. The phylogenetic analysis demonstrated that *A. pintoi* formed a single branch among genus *Arachis*. The whole chloroplast genome of *A. pintoi* will be a useful resource for future studies on phylogeny and conservation in *Arachis.*

*Arachis pintoi* Krapov. & W.C.Greg. is a perennial herb of the genus *Arachis* in Leguminosae (Krapovickas and Gregory 1994). It is widely distributed in tropical and subtropical regions (Paris et al. 2009). *A. pintoi* is also known as wild peanut with a stoloniferous and creeping growth habit. Its leaves are alternate phyllotaxis, each leaf consist of four leaflets. Due to its fast growth rate, long flowering period and high fresh grass yield, *A. pintoi* can be utilized in pure stands under grazing as cut-and-carry forage or as artificial ornamental lawns (Vu et al. 2019). In addition, *A. pintoi* has high crude protein content, good palatability, dense underground rhizomes, abundant nodules, as well as strong barren and trampling resistance (Lascano and Thomas 1988). For these reasons, *A. pintoi* is considered to be a high-quality perennial leguminous forage resource with domestication potential, which is expected to be cultivated and utilized in areas where *M. sativa* and other alfalfa species cannot survive in the winter (Hawton et al. 1990). To facilitate its genetic research and contribute to its utilization, in this study, the complete chloroplast genome of the *A. pintoi* was assembled from the whole genome Illumina sequencing data. Phylogenetic analysis was conducted to be used in further studies on its chloroplast genetic engineering.

Fresh and young leaves of *A. pintoi* were collected from Lingshui County, Hainan Province, China (18°30′27″ N, 110°01′59″ E) for DNA extraction. Voucher specimen was deposited at Zhejiang Academy of Agricultural Sciences with the specimen voucher number of FP200817. Total genomic DNA was extracted from sampled leaves using the Plant Genomic DNA Kit (Tiangen Biotech, Beijing, China) following the manufacturer’s instructions and sequenced based on the Illumina pair-end technology. Then, a total of 49,464,196 raw reads were obtained by next-generation sequencing, conducting on the Illumina Hiseq 4000 Platform (Illumina, San Diego, CA). The complete chloroplast genome was assembled via NOVOPlasty (Dierckxsens et al. 2017) with the chloroplast genome sequence of *Stylosanthes viscosa* (GenBank accession number: NC_039161) used as a reference sequence. The mean sequencing depth was 1030 × for the chloroplast genome of *A. pintoi*. The annotation was performed using an online GeSeq (https://chlorobox.mpimp-golm.mpg.de/geseq.html/), and the gene map of *A. pintoi* chloroplast genome was obtained by using OGDRAW (https://chlorobox.mpimp-golm.mpg.de/OGDraw.html). Finally, a complete chloroplast genome of *A. pintoi* and annotation information had been submitted to GenBank with accession number MW561116.

The complete chloroplast genome of *A. pintoi* is 156,185 bp in length, consists of a large single-copy region (LSC, 85,637 bp), a small single-copy region (SSC, 18,908 bp), and a pair of inverted repeat regions (IRs, 25,820 bp). The overall GC content was 36.4%. The complete chloroplast genome contains 112 unique genes, including 80 protein-coding genes, 28 transfer RNA (tRNA) genes, and four ribosomal RNA (rRNA) genes. Among these genes, fifteen of them contain one intron and three of them contains two introns.

To examine the phylogenetic position of *A. pintoi*, a phylogenetic analysis was performed based on the complete chloroplast genome sequences of 18 Papilionoideae species. All of the chloroplast genome sequences were aligned by using MAFFT (Kazutaka and Standley 2013). Using those whole chloroplast genomes sequences, a phylogenetic tree was constructed by the RAxML-software-embedded method of maximum-likelihood (ML) with 1000 bootstrap replicates (Alexandros et al. 2017) based on conducted adopting Kimura 2-parameter model. *Stylosanthes viscosa* was used as the outgroup. As shown in the ML phylogenetic tree (Figure 1), the results based on chloroplast genome are in agreement with previous morphological and molecular analyses (Wang et al. 2019). The resulting tree showed that *A. pintoi* formed a single branch among genus *Arachis* with 100% bootstrap support value. The results provide valuable information for future studies on phylogenetic and evolution adaptation studies on *A. pintoi* and its related species.

**Figure 1.**
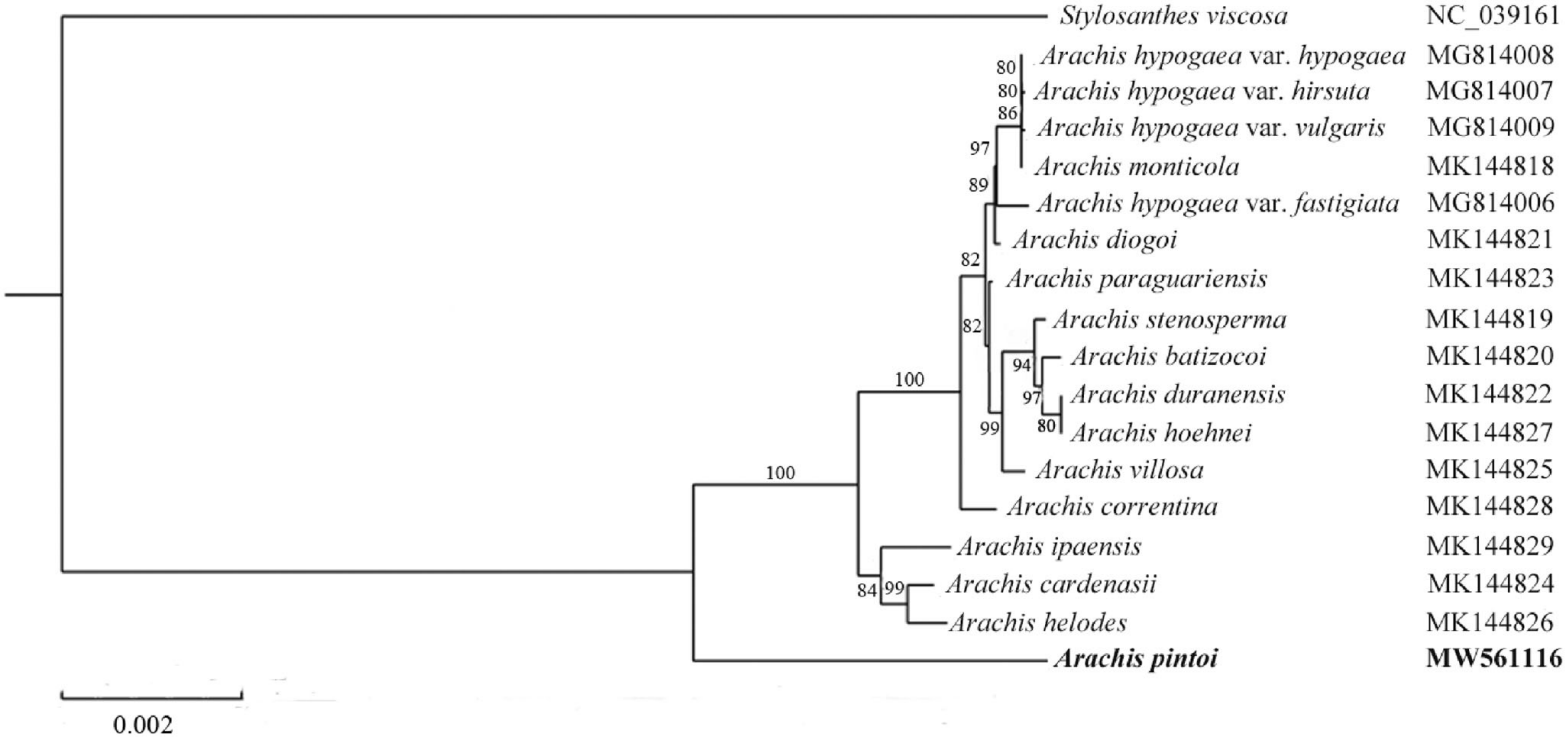
A maximum likelihood (ML) phylogenetic tree based on the 18 species chloroplast genomes was constructed. *Stylosanthes viscosa* was used as the outgroup. The numbers on branches are bootstrap support values.

## Data Availability

The genome sequence data that support the findings of this study are openly available in GenBank of NCBI at (https://www.ncbi.nlm.nih.gov/) under the accession no. MW561116. The associated BioProject, SRA, and Bio-Sample numbers are PRJNA698735, SRR14753916, and SAMN19599397, respectively.
